# Optimizing Early Ophthalmology Clinical Trials: Home OCT and Modeling Can Reduce Sample Size by 20% to 40%

**DOI:** 10.1167/tvst.14.9.2

**Published:** 2025-09-02

**Authors:** Jacques Hermes, Bernhard Steiert

**Affiliations:** 1 Roche Pharma Research and Early Development, Pharmaceutical Sciences, Roche Innovation Center Basel, F. Hoffmann-La Roche Ltd, Basel, Switzerland; 2 Institute of Physics, University of Freiburg, Freiburg, Germany; 3 Freiburg Center for Data Analysis and Modelling (FDM), University of Freiburg, Freiburg, Germany

**Keywords:** home OCT, CST, efficient trial design, mathematical modeling

## Abstract

**Purpose:**

Early-stage clinical trials for major retinal diseases face challenges due to substantial interpatient variability, end points with high intrapatient variability, and prolonged follow-up periods required to detect treatment effects. This study explores whether integrating home optical coherence tomography (OCT) monitoring with pharmacokinetic/pharmacodynamic (PK/PD) modeling can reduce clinical trial sample size.

**Methods:**

A population PK/PD model was developed using longitudinal central subfield thickness data from a home OCT study. Monte Carlo simulations and bootstrapping were used to evaluate the impact of different monitoring strategies on sample size requirements to detect a simulated effect size of approximately 50-µm central subfield thickness reduction over an active comparator drug.

**Results:**

To reliably detect this effect, traditional biweekly in-clinic monitoring required 41 to 54 patients per arm, whereas home OCT monitoring only required 33 to 35 patients per arm, representing a 20% to 40% sample size reduction.

**Conclusions:**

These findings highlight the potential for home OCT and PK/PD modeling to improve trial efficiency and patient convenience while maintaining statistical power.

**Translational Relevance:**

By reducing sample size requirements while maintaining statistical power, this approach can streamline clinical trials, expediting the development of new retinal therapies and improving patient access to treatment.

## Introduction

Increasing complexity of trial designs and the high cost associated with patient recruitment and follow-up represent significant challenges in ophthalmology, where end points require long observation periods.^1^ These challenges are particularly pronounced in retinal diseases such as neovascular age-related macular degeneration (nAMD), where the need for frequent imaging at visits and long follow-up periods further exacerbates the logistical and financial burdens of clinical research. nAMD requires regular monitoring of diseased eyes in adults to guide treatment decisions and prevent under- or overtreatment.^2^ Frequent monitoring and treatment visits can be burdensome for patients, particularly older individuals, leading to logistical challenges in the execution of clinical trials.^2^ The necessity for frequent anti-vascular endothelial growth factor (VEGF) injections—typically every 4 to 8 weeks—alongside regular imaging can contribute to adherence issues and patient dropout in clinical settings.^3^

Recent advances in home-based monitoring technologies, such as home optical coherence tomography (OCT) devices, have the potential to transform clinical trial designs by enabling frequent, patient-operated imaging outside of traditional clinical settings. Home OCT devices have been developed, such as SCANLY by Notal Vision, capable of capturing high-frequency retinal thickness measurements and retinal fluid volumes, providing unprecedented temporal resolution in disease monitoring. These devices have been primarily used for real-world disease monitoring and their application for personalized treatment regimens is being discussed.^4^^,^^5^ However, their application and utility in clinical trial settings remains underexplored.

Pharmacokinetic/pharmacodynamic (PK/PD) models allow for the characterization of drug effects over time and can provide a framework for optimizing study designs with fewer patients while maintaining statistical power. High-frequency longitudinal data from home OCT devices could be combined with advanced mathematical modeling approaches, to potentially improve trial efficiency. By integrating densely sampled home OCT data with a PK/PD model, it should be possible to better quantify treatment effects and reduce the sample size required for robust statistical inference in clinical trials.^6^

In this in silico study, we developed a PK/PD model describing central subfield thickness (CST) dynamics after anti-VEGF treatment in nAMD patients. The model was calibrated using longitudinal home OCT data from a previously published observational study in nAMD patients.^7^ Using Monte Carlo simulations and bootstrapping, we evaluated the impact of different monitoring strategies—traditional on-site visits versus frequent home OCT measurements—on the clinical trial sample size required to detect a certain effect.

## Methods

### Population PK/PD Approach

Population PK/PD modeling describes the variability in drug concentrations and responses among individuals within a target population by incorporating both fixed and random effects.^8^^,^^9^ The structural model defines the typical population behavior using fixed-effect parameters, denoted as θ, and interindividual variability (IIV) is captured by random effects, denoted as η.

For a given individual *i*, a PK/PD parameter *P*_*i*_ (e.g., clearance or volume of distribution) is modeled as

(1) $$\begin{array}{c} P_{i}=\exp\left(\theta \right)\cdot \exp\left(\eta_{i}\right), \end{array}$$

where exp (θ) represents the typical value of the parameter in the population, and exp (η_*i*_) accounts for individual deviations from this typical value. The parameters are log-transformed to avoid negative parameter values and more robust optimization during fitting. The random effects η are assumed to follow a normal distribution with mean zero and variance-covariance matrix Ω, that is,

(2) $$\begin{array}{c} \eta ∼N(0,\Omega ). \end{array}$$

This hierarchical modeling framework allows the estimation of population parameters while accounting for individual differences, thereby improving the predictive power of PK/PD analyses.

### Mathematical Modeling of CST

**Figure 1.**
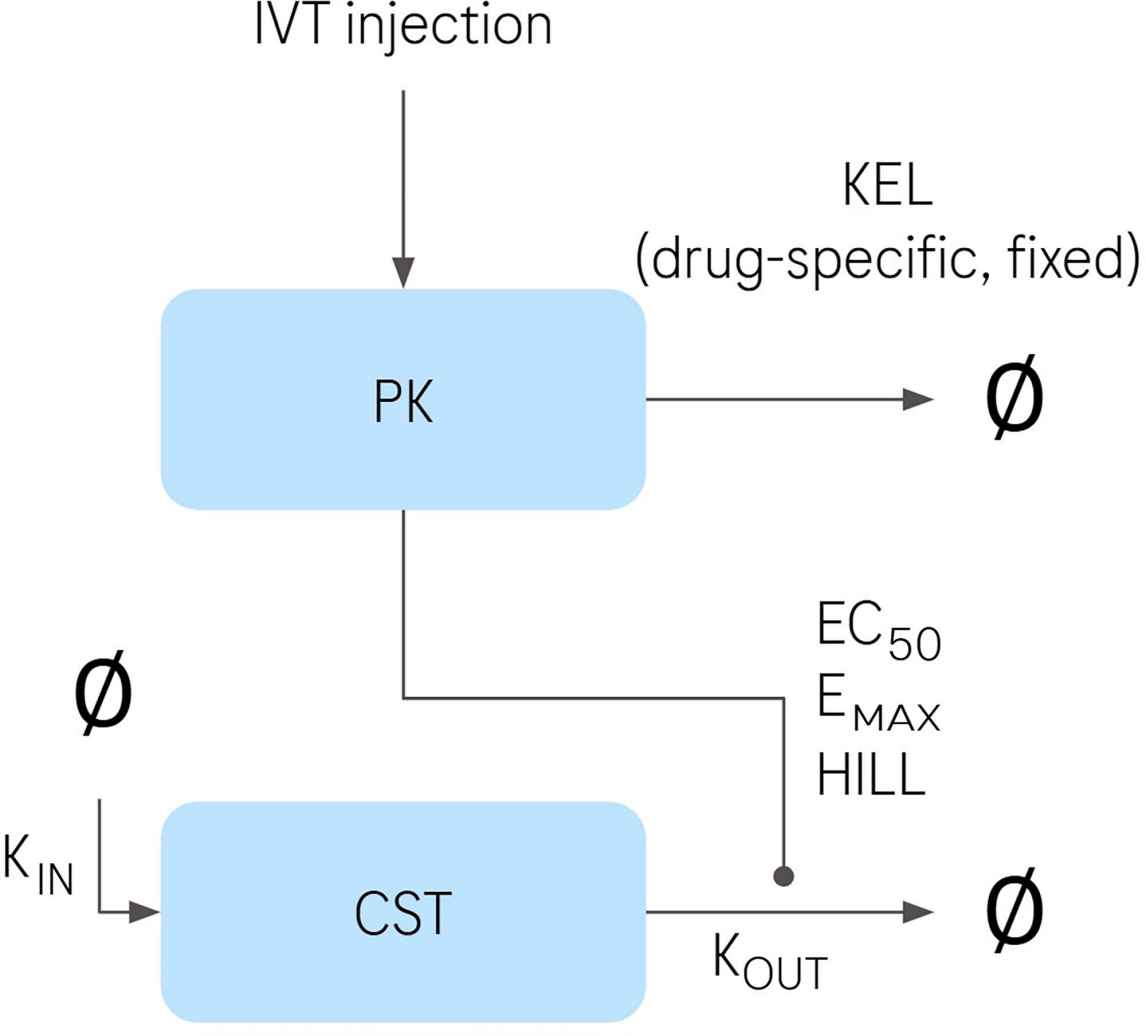
Schematic representation of the two compartment indirect response model. *Light blue boxes* indicate the compartments, and *arrows* indicate flows with annotated corresponding parameters. Flows from or toward empty set symbol indicate zero-order production or first-order degradation, respectively.

A two-compartment indirect response population PK/PD model was developed and implemented in NONMEM.^10^ The two compartments represent the PK compartment and the response variable (CST), which is subject to indirect regulation. A schematic representation of the model is shown in Figure 1. All model parameters were estimated on natural log-scale. The optimization was performed with NONMEM v.7.4.3. and using the FOCEI algorithm.

#### Pharmacokinetic Model

The elimination of the drug was assumed to follow a first-order process

(3) $$\begin{array}{c} \frac{dPK}{dt}=-K_{\text{EL}}\cdot PK \end{array}$$

with an elimination rate constant

(4) $$\begin{array}{c} K_{\text{EL}}=\frac{\ln2}{T_{1/2}}, \end{array}$$

which depends on the administered drug and where *T*_1/2_ is the fixed drug-specific half-life (*T*_1/2_ = 9 days for aflibercept and *T*_1/2_ = 6.5 days for ranibizumab).^11^

The initial PK concentration is denoted as *PK*_0_, modeled as:

(5) $$\begin{array}{c} PK_{0}=\exp\left(\theta_{PK_{0}}+\eta_{PK_{0}}\right). \end{array}$$

#### PD Model

The response variable CST follows an indirect response model with synthesis rate *K*_IN_ and degradation rate *K*_OUT_:

(6) $$\begin{array}{ccc} \;\frac{dCST}{dt} & = & K_{\text{IN}}-K_{\text{OUT}}\\ \; & \times & \left(1+E_{\text{MAX}}\frac{{\left(PK+\varepsilon \right)}^{H}}{EC_{50}^{H}+{\left(PK+\varepsilon \right)}^{H}}\right)CST. \end{array}$$

A small numerical value ϵ = 10^−8^ was added to ensure numerical stability during the calculation of the gradients for very small *PK* values. ϵ has no relevant influence on model fits or predictions. We applied the following reparameterizations

(7) $$\begin{array}{c} K_{\text{OUT}}=\frac{K_{\text{IN}}}{CST_{\text{MIN}}+CST_{\text{DEL}}}, \end{array}$$

(8) $$\begin{array}{c} E_{\text{MAX}}=\frac{CST_{\text{DEL}}}{CST_{\text{MIN}}}, \end{array}$$

with parameters

(9) $$\begin{array}{c} CST_{\text{DEL}}=\exp\left(\theta_{CST_{\text{DEL}}}+\eta_{CST_{\text{DEL}}}\right), \end{array}$$

(10) $$\begin{array}{c} K_{\text{IN}}=\exp\left(\theta_{K_{\text{IN}}}+\eta_{K_{\text{IN}}}\right), \end{array}$$

(11) $$\begin{array}{c} CST_{\text{MIN}}=\exp\left(\theta_{CST_{\text{MIN}}}+\eta_{CST_{\text{MIN}}}\right), \end{array}$$

(12) $$\begin{array}{c} H=\exp(\theta_{H}+\eta_{H}), \end{array}$$

where *CST*_DEL_, *K*_IN_, *CST*_MIN_, and *H* (Hill coefficient) are estimated parameters. The parameters *K*_OUT_ and *E*_MAX_ are derived parameters, which ensures independence between the remaining parameters. Another upside of this reparametrization is that CST values at any point in time cannot decrease below *CST*_MIN_, preventing unphysiologically small values. The magnitude of CST change over time is governed by *CST*_DEL_, meaning the maximum CST value for an individual is given by

(13) $$\begin{array}{c} CST_{\mathrm{MAX}}=CST_{\text{MIN}}+CST_{\text{DEL}}. \end{array}$$

It is assumed the disease progression of the patients is at steady state, meaning with no treatment, a patient will have an increase in CST up to *CST*_MAX_ and even with ideal treatment CST cannot be reduced below *CST*_MIN_.

The drug effect is driven by an *E*_MAX_ model with drug-specific *EC*_50_ values

(14) $$\begin{array}{c} EC_{50,\text{aflibercept}}=\exp\left(\theta_{EC_{50,\text{aflibercept}}}+\eta_{EC_{50}}\right), \end{array}$$

(15) $$\begin{array}{c} EC_{50,\text{ranibizumab}}=\exp\left(\theta_{EC_{50,\text{ranibizumab}}}+\eta_{EC_{50}}\right), \end{array}$$

denoting the concentration of drug in the vitreous, necessary to account for 50% of drug effect at steady state. Here, the two *EC*_50_ parameters share a common random effects distribution ($\eta_{EC_{50}}$). This assumption reflects that, although the mean *EC*_50_ for a given patient differs between two drugs, the overall IIV is comparable between the two drugs.

The initial CST value is denoted as *CST*_0_, modeled as:

(16) $$\begin{array}{c} CST_{0}=\exp\left(\theta_{CST_{0}}+\eta_{CST_{0}}\right). \end{array}$$

#### Error Model

The residual variability for CST, including observational noise, was modeled using an additive error

(17) $$\begin{array}{c} \;Y=CST+\text{ERR},\;\text{where}\;\text{ERR}∼N(0,\sigma^{2}), \end{array}$$

where the parameter σ was also estimated. It is the only model parameter that was estimated on a linear scale.

### Error Propagation

The goal of this in silico study was to determine the required number of patients, *N*, per arm of a hypothetical clinical trial to detect, with 95% certainty, a clinically relevant difference in CST between the trial drug arm and the active comparator arm at week 8 after the last dose.

To quantify the uncertainty of the estimated model parameters and propagate this uncertainty to the state of interest, we used a bootstrap resampling approach followed by Monte Carlo simulations.

#### Bootstrapping

Bootstrapping is a resampling method used to estimate the uncertainty of model parameters by generating multiple resampled datasets from an original dataset.^12^ Each resampled dataset is created by drawing *N* samples with replacement from a larger population, and multiple iterations of this procedure provide variability across datasets and subsequently estimated parameters.

In this work, we generated a population of 1000 simulated patients based on the model and assumed this sample was sufficiently large to represent the complete parameter space relevant for our clinical trial simulation. From this population, we drew *N* patients with replacement and performed model fitting. This resampling and fitting process was repeated 200 times for a range of sample sizes *N*. The distributions of the estimated model parameters obtained from these 200 fits were used to quantify uncertainty, expressed as the standard deviation of the parameter estimates. The resampling was performed without additional constraints to ensure a broad and representative sample.

#### Monte Carlo Simulations

Monte Carlo simulations are a widely used method for uncertainty propagation in nonlinear systems, relying on random sampling from probability distributions.^13^^,^^14^ In this context, Monte Carlo simulations allowed us to propagate the uncertainty in model parameters to the predicted clinical state, CST over time.

Specifically, we sampled 1000 parameter sets from the covariance matrix obtained via bootstrapping of the 200 fits. The parameters were sampled on a log scale, and no further refitting was performed; instead, the sampled parameters were directly used for simulating CST time courses. The resulting distribution of CST values captured the propagated uncertainty due to parameter estimation variability.

### *Z*-Score and Confidence Intervals

To determine the required sample size per arm, we used the *z*-score corresponding with a 95% confidence level. The *z*-score represents the number of standard deviations a data point differs from the mean and is commonly used in hypothesis testing and confidence interval estimation.^15^ In this work, we use it to determine the necessary sample size *N* to obtain a 95% confidence level on a certain clinical endpoint, meaning the sample size for which the *z*-score crosses the threshold value of 1.96.

The *z*-score is calculated as

(18) $$\begin{array}{c} z=\frac{\Delta }{\sqrt{SE_{1}^{2}+SE_{2}^{2}}}, \end{array}$$

where

• Δ is the difference in the mean CST between the trial drug arm and the active comparator arm;
• *SE*_1_ and *SE*_2_ are the standard errors of CST in the trial drug arm and the active comparator arm, respectively; and
• The denominator, $\sqrt{SE_{1}^{2}+SE_{2}^{2}}$, represents the combined uncertainty from both arms.

### Statistical Analysis

Different statistical analysis approaches are used to determine the treatment effect.

(1) In the *classical statistical setting*, the standard error of the end point, here CST, is estimated using a bootstrap approach applied to the arithmetic mean of the individually simulated endpoint CST values at 8 weeks after final treatment across increasing patient numbers.
(2) In the *model-based setting*, we perform the workflows outlined in the Methods section on Error propagation. In the on-site *monitoring setting*, we used the sparsely sampled simulated dataset; in the *home monitoring setting*, we use the densely sampled one.

### Waterfall Plot Analysis

A multistart optimization approach was used to verify the robustness of the estimated global optimum. The objective function values (OFVs) from independent runs, each initialized with different starting conditions, were recorded. These values were then plotted in ascending order to generate a waterfall plot. This visualization allows an assessment of whether the identified global optimum is consistently found as local optimum among multiple fitting runs from random initial values, indicating the absence of additional relevant unidentified minima.^16^

### Profile Likelihood Analysis

To assess parameter uncertainty and evaluate the reliability of parameter estimates, a profile likelihood analysis was conducted.^17^^,^^18^ Each parameter of interest was systematically varied while reoptimizing all other parameters to maintain the best possible fit to the data. The resulting OFV were plotted against the perturbed parameter values and a locally estimated scatterplot smoothing function^19^ was fitted to the sparse profile to obtain a continuous profile. Confidence intervals were derived from the likelihood ratio test, using a threshold corresponding with a predefined significance level (e.g., 95% confidence). This approach provides a direct assessment of parameter identifiability and the potential presence of flat or ill-defined regions in the likelihood surface.

### Prediction-Corrected Visual Predictive Check

A prediction-corrected visual predictive check (pcVPC) was performed to evaluate the predictive performance of the model while adjusting for time-dependent or covariate-related trends in the simulated data.^20^ The prediction correction accounts for variations in the typical predicted response, ensuring a more meaningful comparison between observed and simulated data.

For each observed measurement *Y*_*i*_, a prediction-corrected value $Y_{i}^{\text{pc}}$ was computed as:

(19) $$\begin{array}{c} Y_{i}^{\text{pc}}=Y_{i}\times \frac{{\tilde{Y}}_{\text{median}}^{\text{sim}}}{Y_{i}^{\text{sim}}}, \end{array}$$

where $Y_{i}^{\text{sim}}$ is the simulated counterpart of the observation *Y*_*i*_, and ${\tilde{Y}}_{\text{median}}^{\text{sim}}$ is the median of the simulated values within the corresponding bin. The binning was performed based on time to ensure a sufficient number of observations per bin while maintaining temporal resolution.

After correction, the prediction-corrected observations were compared with the percentiles (e.g., 10th, 50th, and 90th) of the prediction-corrected simulated data. The pcVPC was then visualized by overlaying the corrected observed data with the simulated prediction intervals, allowing for a robust assessment of model adequacy.

### Home OCT Data

The mathematical model introduced in Figure 1 was calibrated using data from a previously published longitudinal study,^7^ in which 15 patients performed near-daily OCT scans of their eyes using the home OCT device developed by Notal Vision. This study provided a densely sampled dataset of CST measurements, with a mean scan frequency of 5.7 ± 0.9 scans per week and a median scan time of 42 seconds. Notably, 93% of all collected scans were eligible for automated analysis using the Notal OCT Analyzer, indicating high adherence and scan quality.

The original study included 15 participants with a mean age of 73.4 ± 6.5 years (range, 57–81 years), of whom 53% were women. Most participants had both eyes eligible, resulting in a total of 29 scanned eyes. Of the 29 eyes included, 83% were diagnosed with nAMD and 17% with intermediate AMD. The mean and median best-corrected visual acuity at enrollment was 20/40 (range, 20/20–20/200), and remained stable throughout the study. Among eyes with nAMD, the mean number of prior anti-VEGF injections at enrollment was 33 ± 28.^7^

Several eyes were excluded from model calibration, as detailed elsewhere in this article. Five eyes were excluded based on clinical classification as early or mild disease—conditions not representative of study eyes typically enrolled in interventional nAMD trials, which could bias model parameter estimates.

During the study, patients received intravitreal (IVT) injections of four different anti-VEGF agents: bevacizumab, brolucizumab, ranibizumab, and aflibercept. Because only a single patient received bevacizumab and another single patient received brolucizumab, these individuals were excluded from analysis, as estimating the *EC*_50_ of a drug based on a single individual was deemed unreliable. Additionally, data from one eye were excluded due to conversion from intermediate AMD to nAMD during the study period—a disease transition not accounted for in the mathematical model.

Ultimately, 20 eyes (17 of which were under active IVT treatment) were used in model calibration, contributing a total of 1628 CST measurements. For further details on the dataset and study protocol, see Liu et al.^7^

## Results

### Model Calibration and Identifiability Analysis

**Figure 2.**
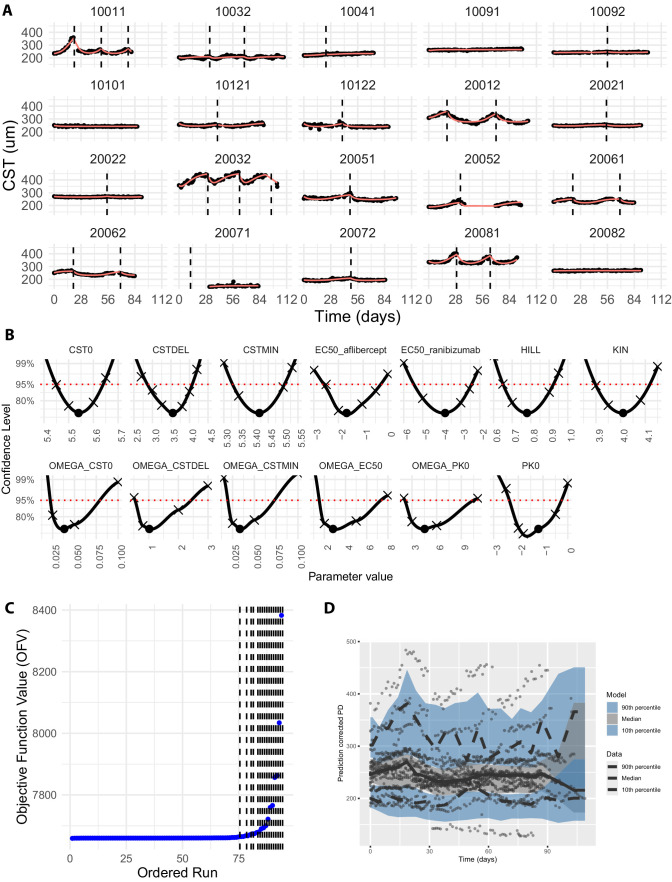
(**A**) Data used for the calibration of the model (*points*) and resulting model fits of the individual predictions (*red lines*). IVT injections are shown via dashed vertical lines. (**B**) Profile likelihood for fitted parameters. The best-fit value of the parameter is indicated by a point, the parameter values for which the profile likelihood was calculated are shown as crosses and the resulting profile likelihood was obtained by fitting a locally estimated scatterplot smoothing function to these values. (**C**) Ordered and numerically sorted OFV obtained via a multistart approach. *Dashed vertical lines* separate steps with a difference in OFV bigger than one, indicating potential local optima. (**D**) Prediction corrected visual predictive check.

The initial version of the model included random effects on all parameters. However, this setup led to numerical instability, with extreme parameter estimates and nonreproducible fits when starting at alternative initial values. To robustify the model, IIVs were systematically removed while monitoring stability and fitting performance. As a result, the random effects on both *H* and *K*_IN_ were removed. The revised model provided reliable individual predictions (Fig. 2A) and adequate overall description of the data as seen in the pcVPC (Fig. 2D).

The final model includes 14 parameters, including an error parameter. The best fit estimates and their associated standard errors are presented in Table.

**Table.** Best-Fit Parameters and Standard Errors

Parameter Name	Units	Best-Fit Value	exp(Best-Fit Value)
*CST*_DEL_	ln (µm)	3.6 ± 0.3	36.6 (27.1–49.4)
*K*_IN_	ln (µm/day)	4.0 ± 0.5	54.6 (33.1–90.0)
*EC*_50,aflibercept_	ln (mg)	−1.7 ± 0.7	0.18 (0.09–0.37)
*EC*_50,ranibizumab_	ln (mg)	−3.8 ± 0.8	0.022 (0.010–0.050)
*CST*_MIN_	ln (µm)	5.42 ± 0.04	225 (217–235)
*CST*_0_	ln (µm)	5.54 ± 0.05	255 (242–268)
*PK*_0_	ln (mg)	−1.2 ± 0.4	0.30 (0.20–0.45)
*H*	ln (unit)	0.78 ± 0.17	2.2 (1.84–2.6)
σ	µm	5.5 ± 0.6	
Ω_1_	unit	1.02 ± 0.36	
Ω_3_	unit	2.8 ± 1.4	
Ω_4_	unit	0.033 ± 0.015	
Ω_5_	unit	0.038 ± 0.017	
Ω_6_	unit	4.5 ± 2.5	

Parameters in the first half of the table are displayed on the natural log-scale, while the second half represents them on a linear scale.

To evaluate model robustness in terms of reproducible fitting results, a multistart analysis was conducted by repeated fitting with varying initial parameter estimates. The OFVs were recorded, sorted and plotted to generate a waterfall plot (Fig. 2C), revealing that most initial conditions converged to the same minimum OFV, suggesting that the above maximum-likelihood estimate is the global optimum.

Furthermore, a profile likelihood analysis confirmed that all model parameters exhibit finite confidence intervals, indicating parameter identifiability (Fig. 2B). The identifiability of the parameters also confirms that the parameters are indeed independent from each other and not strongly correlated, as intended by the reparameterization in Equations (7) and (8) and can thus be sampled independently in a simulation setting.

Finally, to assess goodness-of-fit, diagnostic plots were conducted (Supplementary Figs. S1 and S2). The results indicate that most diagnostic plots, like IPRED vs DV or the distribution of CWRES, exhibited expected behavior, in addition to a suitable pcVPC (Fig. 2D), confirming an adequate model fit and model simulation properties. However, discrepancies were observed in the PRED vs. DV plots, where predicted values using estimated population parameters deviated from the observed data.

This deviation likely stems from two primary factors. First, substantial IIV in CST dynamics was observed, influencing the population mean’s ability to capture individual patient time courses. Second, population-level predictions remained within a narrow range (230–265 µm). This finding may be attributed to the inclusion of eyes in the dataset from^7^ that are not representative of a clinical trial, because the study was an observational study where both eyes were scanned, even if the disease was inactive in one of the eyes exhibiting minimal changes in CST over time. Consequently, the estimated population mean *CST*_DEL_ was relatively small (35 µm), which may not reflect real-world clinical trial scenarios, where often only eyes with active disease that respond stronger to treatment are included.

To address this discrepancy between an observational study of both eyes and an investigational study of selected eyes, a revised population mean of 100 µm was chosen for *CST*_DEL_ in subsequent clinical trial simulations, ensuring a more representative dataset for evaluating treatment effects under realistic conditions.

Given its goodness of fit, robustness and identifiability, this model is considered as the final model and is used for simulating virtual patient cohorts and conducting clinical trial simulations for both approved treatments (aflibercept and ranibizumab) and for predicting hypothetical investigational therapies and/or customized dosing regimens.

### Clinical Trial Simulation

**Figure 3.**
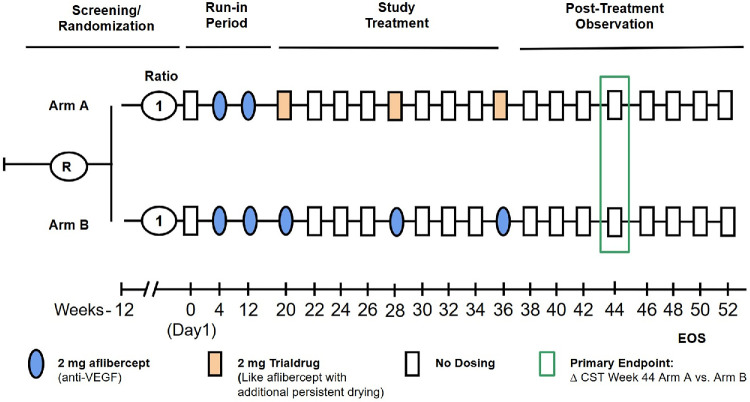
Clinical trial scheme. Horizontal lines depict study arms. EOS = end-of-study, R = randomization. Patients receive two aflibercept 2 mg IVT injections at week 4 and week 12 visits (*blue ellipses*) during the run-in period. Patients in arm A then receive a 2-mg dose of the trialdrug (*orange rectangles*) and patients in arm B continue receiving aflibercept 2-mg injections. During all visits, including those without IVT injections (*open rectangles*), OCT scans are being taken.

Using the calibrated mathematical model, data for the in silico study was then simulated. The simulation followed a hypothetical yet realistic clinical trial scheme illustrated in Figure 3, with two treatment arms: the trial drug (arm A, orange) and an active comparator drug, here aflibercept (arm B, blue). The data were generated under two monitoring conditions: a high-frequency home monitoring setting with five or six CST measurements per week and a traditional on-site setting with biweekly CST measurements.

Both treatment cohorts shared identical population means and IIV for the model parameters, except that the trial drug was assumed to result in a 40 µm reduction in the population mean for *CST*_MIN_, compared with aflibercept, that is, *CST*_MIN,aflibercept_ = 226 µm and *CST*_MIN,Trialdrug_ = 186 µm.

Patient-specific parameters were sampled from the distribution estimated in Model Calibration and Identifiability Analysis, with means corresponding with the estimated population values θ and variances Ω reflecting the estimated IIV. These parameters were then used to forward-simulate the mathematical model described in Mathematical Modeling of CST. Random noise, drawn from a normal distribution with the standard deviation σ = 5.5 µm (as estimated in Model Calibration and Identifiability Analysis), was added to the simulated CST values to replicate the residual error, including observational noise.

**Figure 4.**
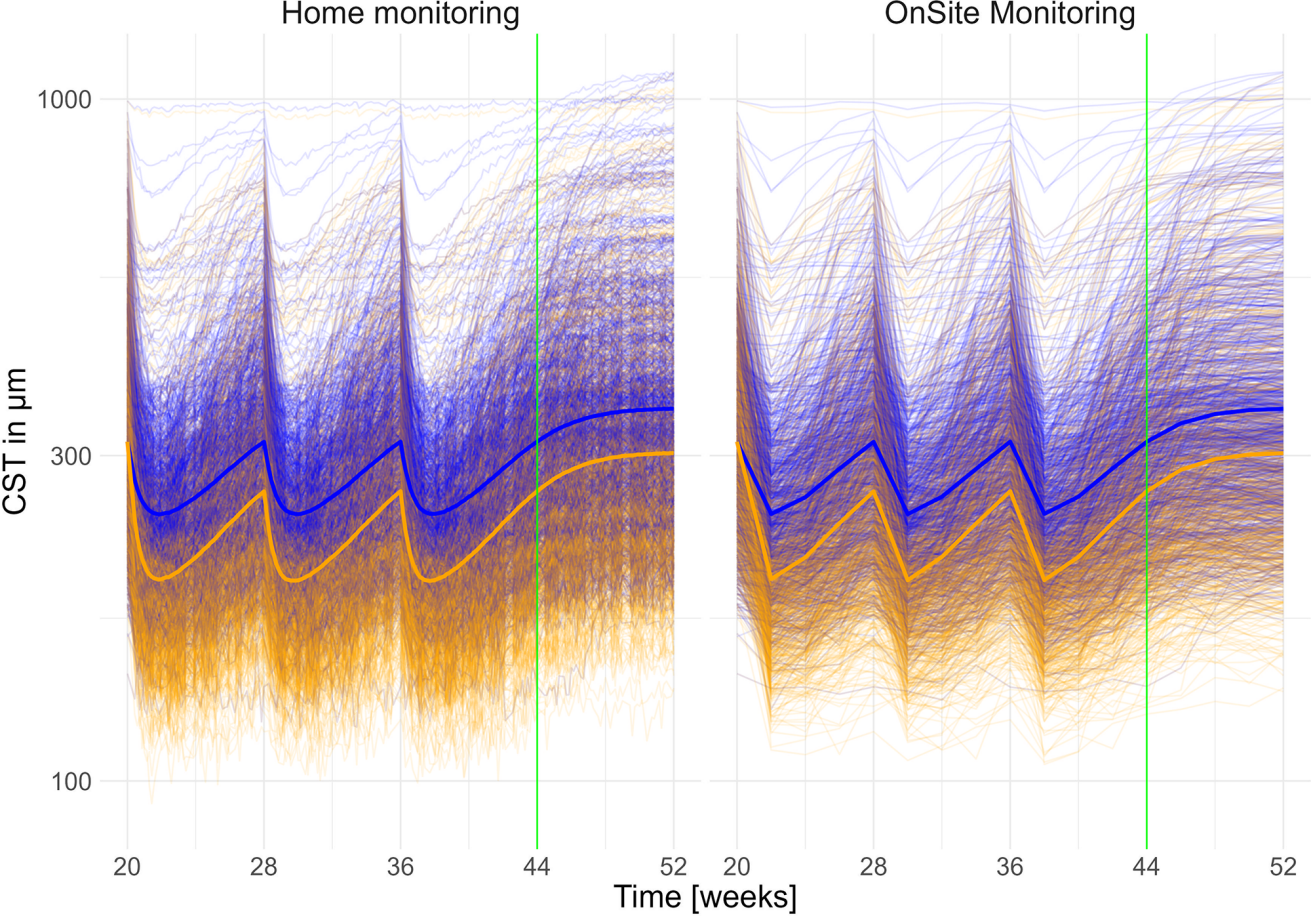
Simulated data for 1000 patients. The simulation was performed according to the clinical trial scheme in Figure 3 with arm A (trial drug arm) in *orange* and with arm B (aflibercept arm) in *blue*. The data were simulated in two settings: The densely sampled home monitoring setting (*left*) with five to six measurements per week and the sparsely sampled on-site setting (*right*) with measurements every 2 weeks according to the visits in the trial scheme. The *green line* marks the clinical end point at week 44. Only data up to this point are used for fitting.

The resulting dataset for 1000 patients per arm is visualized in Figure 4. A clear distinction between the trial drug (orange) and aflibercept (blue) treatment arms is observed, with the trial drug achieving a superior outcome in terms of CST reduction. The effect size is quantified as a mean CST reduction of 48 µm at week 8 after the final dose (week 44). Furthermore, the simulated patient time courses exhibit a broad range of realistic CST dynamics, suggesting that the model-generated virtual cohort effectively represents the diversity of a real-world nAMD patient population.

### Error Propagation Study

**Figure 5.**
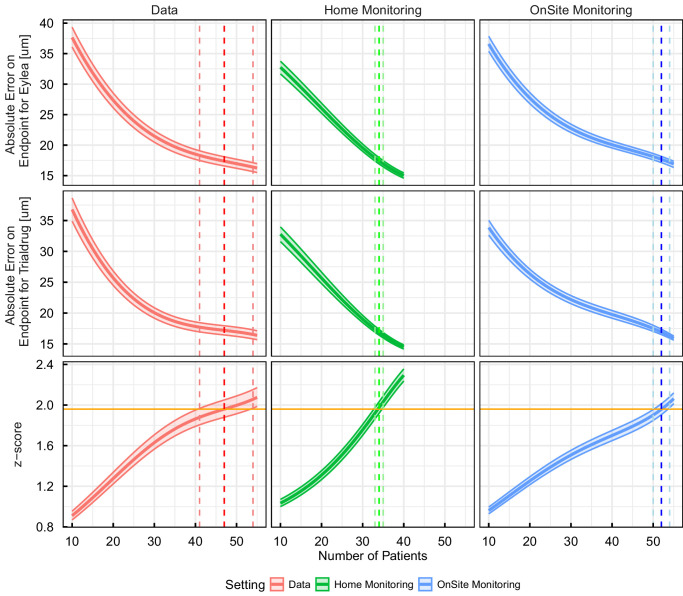
Absolute error on end point measurements (mean CST at week 44) for aflibercept and trial drug across different patient numbers and monitoring settings. The figure presents three monitoring conditions: data, that is, classical statistical setting (*left, red*), home monitoring (*middle, green*), and onsite monitoring (*right, blue*). The *top and middle rows* show absolute errors in micrometers for aflibercept and trial drug, with the 2.5th and 97.5th quantiles shown as ribbons around the estimated standard errors. The *bottom* row depicts the corresponding *z*-scores. The middle dashed vertical lines indicate the number of patients required for the *z*-score to exceed 1.96, whereas the *lighter dashed lines* indicate the zscores obtained using the 2.5th and 97.5th quantiles of the errors.

The error propagation analysis was conducted following the methodology outlined in Error Propagation, with results presented in Figure 5. For both the classical statistical setting and the modeling setting, a bootstrapping procedure with replacement was performed to estimate the variance on the estimated standard error. The 2.5th and 97.5th quantiles of those are shown as ribbons in Figure 5.

The findings indicate that for both the classical statistical setting (“Data” in Fig. 5) and the model-based on-site monitoring setting, the minimum number of patients required to detect the desired effect size with statistical significance is similar, namely 49 (41–54) for the classical statistical method and 52 (50–54) for sparse sampling. With sparse data, the gains that may result from using a modeling approach seem to be compensated by the uncertainty resulting from the sparse data. Therefore, no net efficiency gain over a classical statistical approach can be observed. This classical setting is subsequently referred to as the baseline, where an estimated 50 (41–54) patients per arm are necessary to detect the assumed effect under the assumed variability and observational noise.

In contrast, when mathematical modeling is combined with high-frequency home monitoring as shown in the home monitoring setting, the required sample size is reduced to only 34 (33–35) patients, representing a 20% to 40% reduction in trial size. This reduction translates into substantial resource saving potential and highlights the efficiency gains achievable through the integration of home OCT data with advanced modeling techniques.

## Discussion

The results of this study highlight the potential of integrating home OCT monitoring with PK/PD modeling to improve the efficiency of clinical trials in ophthalmology. By leveraging high-frequency longitudinal data, this approach enables a significant reduction in the required sample size to detect a certain differential effect on CST while maintaining statistical rigor. This finding may have important implications for the design of future trials, particularly in diseases such as nAMD, where treatment response varies widely among patients.

One of the key advantages of home OCT monitoring is its ability to capture disease dynamics with higher temporal resolution than traditional in-clinic visits. This frequent sampling allows for a more detailed understanding of treatment effects and disease progression, reducing uncertainty in model predictions. As demonstrated in this study, this improved data granularity translates directly into smaller required patient cohorts, leading to considerable resource savings in clinical trial execution.

However, several limitations and considerations must be acknowledged. The model calibration was based on a relatively small observational dataset, which may not fully reflect the diversity or characteristics of active trial populations. Although this cohort provides rich longitudinal data, it represents a relatively small and demographically uniform sample. As such, factors such as health literacy, comfort with technology, and clinical diversity may influence the generalizability of the findings and should be addressed in future validation efforts. To partially address the limitations of the dataset, we adjusted the mean *CST*_*DEL*_ value in our simulations to reflect more realistic treatment dynamics as observed in larger interventional studies. Although this approach helps to align the model behavior with expected clinical responses, it introduces an additional assumption that should be interpreted with caution. It is likely that the necessary sample size depends on the *CST*_*DEL*_ of the analyzed population. However, a reduction in the sample size through the use of the presented approach is expected for any value of *CST*_*DEL*_. Future work using broader datasets for model calibration will improve generalizability and ensure robustness across different patient populations and disease phenotypes.

Another limitation is the reliance on patient-operated imaging, which raises valid concerns about adherence and data quality. In our model, we approximated adherence by simulating OCT acquisition on a randomized five to six measurements per week schedule, consistent with adherence levels observed in the original observational dataset. Although real-world variability in image acquisition quality, device use, and patient compliance could introduce noise that affects model predictions, previous observations suggests that the impact of these factors may be limited. Recent studies have demonstrated that patient-operated home OCT devices produce clinically useful data in terms of layer thickness and fluid volume with acceptable image quality and consistency across time.^21^^,^^22^ Nevertheless, these effects were not explicitly modeled and warrant further exploration in future work.

To that end, although this was an in silico study, we propose a specific validation strategy: integrating home OCT into an actual clinical trial where trial size calculations are initially based only on classical visit-based endpoints. In such a design, the observed treatment effect could first be evaluated using the conventional sparse dataset. The same effect could then be tested using the densely sampled data from home OCT, comparing the statistical power and sample size requirements retrospectively. This would allow for benchmarking the in silico-predicted sample size reductions against actual clinical outcomes. Moreover, such a study could directly assess the practical impact of variability in home OCT adherence and data quality on trial endpoints and modeling performance.

Despite these challenges, the integration of home OCT and mathematical modeling represents a promising step forward in ophthalmology clinical research. This study provides a concise foundation for future work investigating how these methodologies can be further refined and applied to other retinal diseases. One such direction is to explore whether the increased information density from high-frequency sampling could allow not only for smaller trial sizes but also for reduced overall trial durations, enabling earlier decision-making. As technology advances and home monitoring devices become more widely available, their incorporation into trial designs may become a standard practice, paving the way for more efficient and patient-centric clinical trials.

## Supplementary Material

Supplement 1

## References

[1] Wong TY and Sabanayagam C. Strategies to tackle the global burden of diabetic retinopathy: from epidemiology to artificial intelligence. *Ophthalmo.* 2020; 243(1): 9–20. 10.1159/000502387 31408872

[2] National Institute for Health and Care Excellence (NICE). Age-related macular degeneration: monitoring late AMD (wet active), 2023. Accessed February 17, 2025.

[3] NHS England. Decision support tool: making a decision about wet agerelated macular degeneration, 2022. Accessed February 17, 2025.

[4] Schneider EW, Heier JS, Holekamp NM, et al. Pivotal trial toward effectiveness of self-administered oct in neovascular age-related macular degeneration. report 2—artificial intelligence analytics. *Ophthalmol Sci.* 2025; 5(2): 100662. 39811265 10.1016/j.xops.2024.100662 PMC11731483

[5] Heier JS, Liu Y, Holekamp NM, et al. Clinical use of home oct data to manage neovascular age-related macular degeneration. *J Vitreoretinal Dis.* 2024: 24741264241302858, doi:10.1177/24741264241302858. PMC11625398 39654701

[6] Dolar-Szczasny J, Drab A, and Rejdak R. Homemonitoring/remote optical coherence tomography in teleophthalmology in patients with eye disorders—a systematic review. *Front Med.* 2024; 11: 1442758. 10.3389/fmed.2024.1442758 PMC11540653 39512616

[7] Liu Y, Holekamp NM, and Heier JS. Prospective, longitudinal study: daily self-imaging with home oct for neovascular age-related macular degeneration. *Ophthalmol Retina.* 2022; 6(7): 575–585. 35240337 10.1016/j.oret.2022.02.011

[8] Sheiner LB, Rosenberg B, and Marathe VV. Forecasting individual pharmacokinetics. *Clin Pharmacol Ther.* 1977; 21(3): 249–253. 10.1002/cpt1979263294 466923

[9] Sheiner LB and Steimer J-L. Pharmacokinetic/pharmacodynamic modeling in drug development. *Annu Rev Pharmacol Toxicol.* 1992; 31(1): 161–191. 10.1146/annurev.pharmtox.40.1.67 10836128

[10] Bauer RJ . Nonmem tutorial part II: estimation methods and advanced examples. *CPT Pharmacometrics Syst Pharmacol.* 2019; 8(8): 538–556. 31044558 10.1002/psp4.12422 PMC6709422

[11] Caruso A, Füth M, Alvarez-Sánchez R, et al. Ocular half-life of intravitreal biologics in humans and other species: meta-analysis and model-based prediction. *Mol Pharm.* 2020; 17(2): 695–709. 31876425 10.1021/acs.molpharmaceut.9b01191

[12] Efron B and Tibshirani RJ. *An introduction to the bootstrap*. New York: Chapman and Hall/CRC; 1994.

[13] Metropolis N and Ulam S. The Monte Carlo method. *J Am Stat Assoc.* 1949; 44(247): 335–341. 18139350 10.1080/01621459.1949.10483310

[14] Robert C and Casella G. *Monte Carlo Statistical Methods. Springer texts in statistics*. New York, NY: Springer; 2010.

[15] Casella G and Berger RL. *Statistical Inference. Duxbury advanced series in statistics and decision sciences*. Stamford, CT: Thomson Learning; 2002.

[16] Tönsing C, Timmer J, and Kreutz C. Optimal paths between parameter estimates in non-linear ode systems using the nudged elastic band method. *Front Phys.* 2019; 7: 1–14.

[17] Maiwald T, Hass H, Steiert B, et al. Driving the model to its limit: profile likelihood based model reduction. *PLoS One.* 2016; 11(9): 1–18. 10.1371/journal.pone.0162366 PMC5010240 27588423

[18] Wieland F-G, Hauber AL, Rosenblatt M, Tönsing C, and Timmer J. On structural and practical identifiability. *Curr Opinion Syst Biol.* 2021; 25: 60–69.

[19] Jacoby WG . LOESS: a nonparametric, graphical tool for depicting relationships between variables. *Electoral Studies.* 2000; 19(4): 577–613.

[20] Bergstrand M, Hooker AC, EWallin J, and Karlsson MO. Prediction-corrected visual predictive checks for diagnosing nonlinear mixedeffects models. *AAPS J.* 2011; 13(2): 143–151. 21302010 10.1208/s12248-011-9255-z PMC3085712

[21] Holekamp NM, de Beus AM, Clark WL, and Heier JS. Prospective trial of home optical coherence tomography-guided management of treatment experienced neovascular age-related macular degeneration patients. *Retina.* 2024; 44(10): 1714–1731. 39287534 10.1097/IAE.0000000000004167 PMC11398287

[22] Blinder KJ, Calhoun C, MG, et al. Home oct imaging for newly diagnosed neovascular agerelated macular degeneration: a feasibility study. *Ophthalmol Retina.* 2024; 8(4): 376–387. 37879537 10.1016/j.oret.2023.10.012 PMC10997472

