# Supplementary material for: Optimizing Early Ophthalmology Clinical Trials: Home OCT and Modeling Can Reduce Sample Size by 20% to 40%
Source: Transl Vis Sci Technol. 2025 Sep 2;14(9):2. doi: 10.1167/tvst.14.9.2 (PMC12410251; doi:10.1167/tvst.14.9.2)
Supplement: Supplement 1 [file tvst-14-9-2_s001.pdf]

A Goodness of fit plots

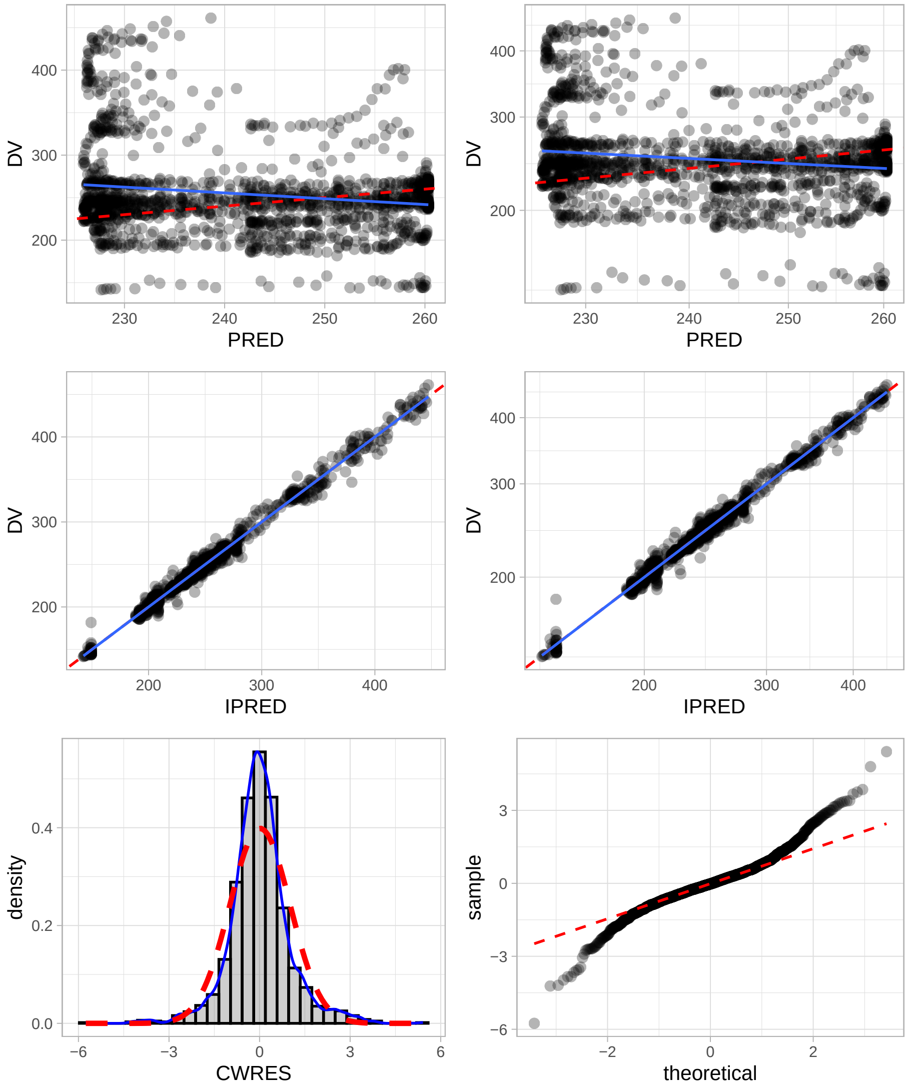

Figure 1: CWRES = conditional weighted residual; DV = dependent variable (CST in um); IPRED = individual predictions (um); PK = pharmacokinetic; PRED = population predictions (um).

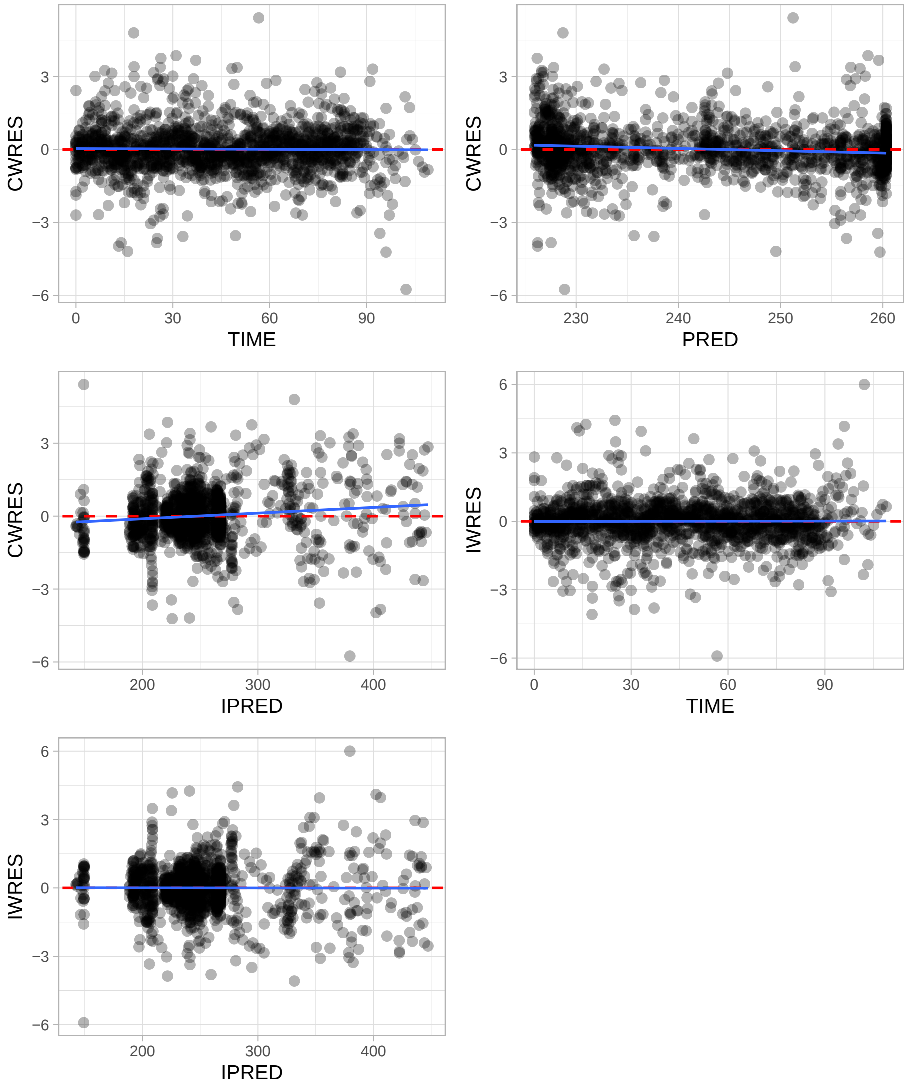

Figure 2: CWRES = conditional weighted residual; DV = dependent variable (CST in um); IPRED = individual predictions (um); IWRES = individual weighted residual; PK = pharmacokinetic; PRED = population predictions (um); TIME = time after first observation (days).
